# Risks to the Unborn: An Umbrella Review on the Effects of Prenatal Maternal Stress Caused by Natural Disasters

**DOI:** 10.1002/smi.70108

**Published:** 2025-09-28

**Authors:** Kaia A. Bustnes, Sarah Schäfer, Linus Held, Hannah Wessels, Maximilian A. Friehs

**Affiliations:** ^1^ Martin‐Luther‐Universität Halle‐Wittenberg Halle (Saale) Germany; ^2^ Rechtspsychologisches Forum Münster Germany; ^3^ Psychology of Conflict Risk and Safety University of Twente Enschede the Netherlands; ^4^ School of Psychology University College Dublin Dublin Ireland

**Keywords:** cortisol, developmental risk factors, disasters, neurological effects, prenatal stress

## Abstract

Traditionally, to promote an optimal pregnancy trajectory and child development, encompassing both physical and mental health, a preventative focus is crucial and ‐ ideally ‐ exposure to negative influences is supposed to be limited. However, when prevention is not feasible, early identification of developmental impairments is paramount to address potential risk factors for future development. Specifically, one source of developmental impairment is prenatal maternal stress. This umbrella review integrates and summarizes current research on the diverse developmental consequences of prenatal maternal stress caused by natural disasters. The cumulative evidence strongly suggests that the effect of maternal stress during pregnancy does not end after pregnancy but can lead to a wide range of detrimental effects on a child's development throughout the whole lifespan. By synthesizing previous empirical findings, the current review provides an overview about potential congenital developmental difficulties as well as the interdependence of these negative effects. The depicted results highlight a risk of overarching negative effects of prenatal stress for the child. It is stated that in order to prevent possible long‐lasting effects this risk has to be effectively taken into account. Possible recommendations for prevention interventions are discussed.

## 1 Introduction

Most people would agree that maximizing opportunities for children's development is important for a sustainable and prosperous future. Extensive support including parental, medical, political, institutional, and social interventions are crucial for fostering stable mental and physical health. While these efforts play a significant role, they may not fully remediate pre‐existing damage. Therefore, a cornerstone of child healthcare is preventive intervention to minimize such harm. Importantly, this includes recognition of potential harm occurring even before birth. In detail, extensive research has demonstrated that prenatal stress can influence various developmental steps resulting in various negative effects for the offspring. Prenatal stress, in that context, refers to the physiological and psychological strain experienced by a pregnant individual, which is also experienced by the unborn child.

### 1.1 Prenatal Stress and Early Human Development

From an evolutionary perspective, the foetus develops in such a way that it can cope in its future environment as best as possible. The prenatal period is considered to be a developmental phase which is especially sensitive to environmental factors. By being responsive to their mothers' conditions before birth, foetuses are supposed to be prepared for their lives after birth—forecasted by cues available during pregnancy, the so‐called *predictive adaptive response* (Bateson et al. 2004; Gluckman et al. 2005). The basic assumption is that physiological systems—like the brain in particular—are highly flexible at this early stage meaning that nerve connections are newly formed and rearranged depending on their use. Thus, foetuses are highly receptive to any prenatal cues and the resulting effects can be long‐lasting and far reaching—positive, but also negative ones. Specifically, on the one hand, the prenatal neural plasticity encourages rapid structural changes. Thereby, stress‐related cues during pregnancy strengthen the child's stress response by claiming it already during this adaptive developmental phase. Thus, prenatal stress exposure also results in an increased resilience to stress. However, on the other hand, neural plasticity also goes in line with an increased vulnerability. For example the negative effect of alcohol, cigarette, and drug consumption during pregnancy on the unborn child has been well researched and is no longer controversial (Avşar et al. 2021; Mamluk et al. 2017; Sundermann et al. 2019; Zhang et al. 2022). Moreover, aetiology models suggest that there are pre‐, peri‐, and postnatal influences affecting the mental as well as physical development over a lifespan, indicating that an increased risk of suffering from specific diseases in adulthood is already being set in the womb (Buss et al. 2012).

According to the underlying mechanisms of far‐reaching effects of prenatal influences, various studies demonstrate an effect of prenatal influences on genomic transcription, that is epigenetic effects. In detail, different studies demonstrate an effect of various prenatal influences ‐ such as maternal pregnancy‐related anxiety, maternal prenatal depression, or parental post‐traumatic stress disorder during pregnancy ‐ on the methylation of the promoter region of the glucocorticoid receptor (NR3C1), the receptor for the stress hormone cortisol (Glover et al. 2018). Besides that, although the human body is capable of adapting to stressors, any adaptation and response to acute stress is linked to numerous changes in the body including cardiovascular regulation, energy metabolism, and immune functioning (Ravi et al. 2021; Selye 1976). As the very first living space, the placenta develops specifically and individually for each pregnancy and is the only communication and exchange possibility between the mother and her baby (Wadhwa et al. 2011). Connected by the umbilical cord, mother and baby form one common blood circulation. Hence, if a pregnant woman is stressed, cortisol flows through her bloodstream. Importantly though, 11β‐Hydroxysteroid‐Dehydrogenase type 2 (11ß‐HSD‐2), an enzyme in the placenta, ensures that the mother's cortisol level does not unhindered pass into the child's bloodstream. Approximately 80%–90% of maternal cortisol is inactivated before reaching the child (Rakers et al. 2020) meaning that only 10%–20% achieve the child. However, animal and human studies indicate that this barrier function is lowered and the activity of 11ß‐HSD‐2 is down‐regulated when the maternal stress level is permanently increased (Jensen Peña et al. 2012; O’Donnell et al. 2012). As a consequence, a child's stress level can significantly be raised by external factors already during pregnancy. Moreover, maternal cortisol can affect the development of the foetal hypothalamic‐pituitary‐adrenal axis. As it changes its activity and alters its sensitivity level, this causes long‐lasting postnatal consequences (Moisiadis and Matthews 2014; Rakers et al. 2020). Maternal stress, along with malnutrition and maternal immune‐related factors, is one of the strongest early environmental factors that can influence the development and health of offspring later in life (Van Den Bergh et al. 2020).

However, so far, effects of prenatal maternal stress have been investigated in predominantly Caucasian samples from high‐income countries (Glover et al. 2018). Yet, it is very likely that many children are affected in low‐ and middle‐income countries as well because of a high risk of stress due to for example wars, interpersonal violence, poverty, or potential infections (Glover et al. 2018).

### 1.2 Natural Disasters as an Equalizing, External Stressor

At the time of writing this manuscript the western media landscape is filled with constant news on the 2025 Palisade Fires in California (USA), in which already many people have lost their lives and livelihood. While the debate about the exact reason for the fire continues, one thing is clear: climate change is at least partially to blame (Goss et al. 2020; Mansoor et al. 2022). Similarly, although potential problems in public administration and infrastructure did not help in overcoming or even avoiding the 2024 Spanish Floods, climate change also played a causal role. Although not all people may agree on all climate change related issues, most people would arguably agree that such disasters have long‐lasting negative consequences for the affected area and its people. Research shows that climate change is linked to an increase in the frequency and intensity of natural disasters, that is human emissions of greenhouse gases are altering the climate leading to more extreme weather events such as heatwaves, floods, and hurricanes (Banholzer et al. 2014; Van Aalst 2006). Observations since 1950 indicate a rise in extreme weather events, with predictions of further increases in the 21st century, including more frequent heatwaves and intense droughts (Banholzer et al. 2014; Coronese et al. 2019). The socio‐economic damages from natural disasters have been rising, with climate change contributing to this trend by increasing the intensity of such events (Coronese et al. 2019); affecting vulnerable populations disproportionately (Benevolenza and DeRigne 2019; Ibarrarán et al. 2009). Thus, if natural disasters cannot be prevented or contained in a timely manner, there is a distinct need for risk prevention and damage mitigation in order to enhance the societal resilience and stress resistance (Benevolenza and DeRigne 2019; Botzen and Van Den Bergh 2009; O’Brien et al. 2006).

Especially, for the most vulnerable among the victims of such disasters, the consequences can be life‐altering. One such vulnerable group are pregnant individuals ‐ the focus of this umbrella review. In that regard, natural disasters provide an opportunity to study the impact of prenatal stress with a largely independent random stressor. Hence, investigating the effects of exposure to natural disasters during pregnancy allows for an investigation of consequences of a stressor which the woman does not influence herself either by behavioural or genetic means, which then could have its own effects. Specifically, because exposure to natural disasters can occur to any person, natural disasters act somewhat like ‘natural experiments’, offering a form of approximately random assignment similar to controlled studies (Cao‐Lei et al. 2014; King et al. 2012; King and Laplante 2015). Importantly, however, one needs to state that exposure to natural disasters depends on for example socioeconomic status, ethnicity, housing conditions, and disability status (see e.g., Jardine et al. 2021; Thomson et al. 2021). Above that, resilience to disaster‐related stress is different between people meaning that the stress is perceived and coped differently by different people (Alves et al. 2021; Hajure et al. 2024; Langley‐Evans et al. 2022; SmithBattle and Phengnum 2023; Tuthill et al. 2021; Walker et al. 2022). In detail, resilience or vulnerability to stress ‐ due to any kind of stressor ‐ is different. Psychological factors such as emotional flexibility, locus of control, social problem solving, and cognitive skills, but also biological factors and their interplay with psychological and environmental factors contribute to stress resilience resulting in an inter‐ and intra‐individually pronounced resilience to stress in a particular situation (van der et al. 2013). However, using natural disasters to study the effects of prenatal maternal stress allows for the examination of the effects of stress on pregnant women and their offspring without the ethical concerns of inducing stress artificially. Moreover, even if different stress resilience does play a role, natural disasters affect broad populations simultaneously and the exposure to this particular stressor is less confounded with person‐specific factors than the exposure to other stressors like financial hardship or experience of physical violence. Thus, investigating the effects of stress due to exposure to natural disasters helps in isolating the effects of stress from person‐specific parameters as best as possible. Furthermore, this approach allows for the differentiation between objective stress (measurable hardship) and subjective stress (reported individual stress), providing insights into how each type affects foetal development during pregnancy and child development after birth (Cao‐Lei et al. 2014; King et al. 2012; St‐Pierre et al. 2018). Moreover, since the timing of a disaster is well documented and the time of exposure to the stressor is better assessable than for other stressors (like e.g., for stress due to financial hardship), the effects of stress at different stages of pregnancy can be investigated to a relatively large extent and tracking developmental outcomes over time in a longitudinal way is more precise (Heynen et al. 2023; Lafortune et al. 2021; Lequertier et al. 2019; Moss et al. 2017). For those reasons, in the current review in particular the effects of prenatal maternal stress caused by exposure to natural disasters are summarized.

### 1.3 The Scope of the Present Review

Experiencing a natural disaster in general is already potentially traumatizing but the consequences may be even more severe if the event coincides with a pregnancy. In the following, we summarize studies which report significant effects of a mother's exposure to a natural disaster on developmental variables in the child. When we talk about prenatal maternal stress below, we refer to severe or persistent stress which goes beyond everyday stress, that is goes beyond stress due to a busy everyday life or a stressful working day.

## 2 Methods

For this umbrella review, we entered four search strings in Scopus and PubMed to get an overview of relevant reviews as well as meta‐analysis concerning the effects of natural disaster related to prenatal maternal stress (PNMS) and different pregnancy outcomes (for an overview of all results, see Table 1).

**TABLE 1:** Search strings and number of results.

Search string	Database	No. Of results
(‘Prenatal stress’) AND (‘disaster’) AND (‘review’)	PubMed	33
(‘Prenatal stress’) AND (‘disaster’) AND (‘meta‐analysis’)	PubMed	4
(‘Maternal stress’) AND (‘disaster’) AND (‘meta‐analysis’)	PubMed	7
(‘Maternal stress’) AND (‘disaster’) AND (‘review’)	PubMed	70
(‘Prenatal stress’) AND (‘disaster’) AND (‘review’)	Scopus	15
(‘Prenatal stress’) AND (‘disaster’) AND (‘meta‐analysis’)	Scopus	4
(‘Maternal stress’) AND (‘disaster’) AND (‘meta‐analysis’)	Scopus	12
(‘Maternal stress’) AND (‘disaster’) AND (‘review’)	Scopus	24

**FIGURE 1:**
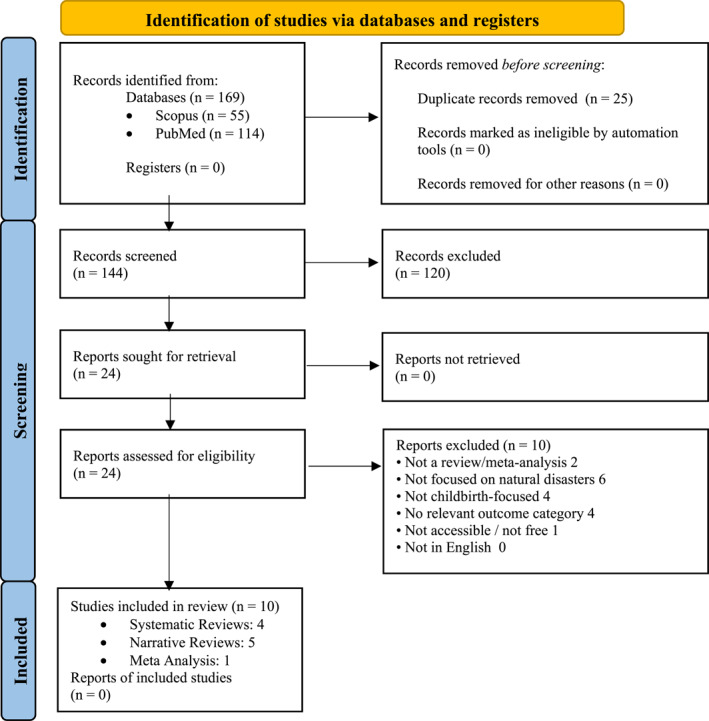
PRISMA flow diagram of the selection process.

To select relevant articles for this review, in the identification stage of the search, we screened 169 articles resulting from the search string. Based on title and abstract, 120 articles were excluded, as they were either duplicates, no reviews or meta analysis, emphasised PNMS unrelated to natural disasters (i.e., man made disasters etc) or other issues such as postnatal care in disaster areas. After screening title and abstract of each remaining article, we selected 24 articles for further screening based on their best contextual fit, whereby none of the authors of this review was involved in any of the selected studies, providing any conflict of interest. We used the PICO framework to define the inclusion criteria for the review as follows: P (population): Women who were pregnant at the time of a natural disaster; I (intervention): prenatal exposure to a natural disaster (e.g., earthquakes, flood, ice storm); C (comparison): developmental outcomes of children whose mothers were not exposed to natural disasters during pregnancy or no comparison; O (outcome): child developmental outcomes (e.g., weight outcomes, gestational duration, cognitive development). Therefore, we considered only internationally published meta‐analyses and reviews focusing on pregnancy outcomes in humans; articles related to man‐made disasters such as wars or nuclear accidents and those that had no free access were excluded. There was no restriction in the publication date of the selected reviews and analysis. After screening the 24 selected articles more thoroughly based on the outlined inclusion and exclusion criteria, we finally selected 10 meta‐analysis and reviews (see Figure 1).

The reported search method resulted in 10 studies, which built a comprehensive overview of methodologically valid studies reporting possible effects of PNMS on a child's development (for a detailed overview of studies see Table 2). Several reviews report the same primary studies. This content overlap was documented and taken into account in order to avoid bias caused by evidence being counted repeatedly.

**TABLE 2:** Overview of meta‐analyses or reviews and their characteristics.

Author	Sample size	Study design	Disaster	Predictors measured in mothers	Outcome categories	Direction and strength
Lafortune et al. (2021)	30 Studies (*n* = 4.556)	Mainly prospective (29/30)	Floods, earthquakes, ice storms, cyclone	Objective stress, subjective stress, psychological stress	Size and weight outcomes, Cognitive and brain development, Duration of gestation, Behavioural outcomes	25 Effect sizes,^a^ 58 Effect sizes,^b^ 25 Effect sizes,^a^ 46 Effect sizes,^a^
Lamichhane et al. (2020)	15 Studies (*n* = 149,866)	Mainly prospective cohort	Ice storm, floods	Objective stress, subjective stress, Physiological measures	Size and weight outcomes	15 Studies,^b^
Amjad et al. (2021)	8 Studies (*n* = 1,702,252)	Mainly retrospective cohort	Wildfires	Objective stress	Size and weight outcomes, Duration of gestation, Infant mortality	7 Studies,^b^ 4 Studies,^c^ 1 Study,^a^
Kyle and Dumitriu (2021)	22 Studies (*n* = not reported)	Not reported	Pandemic (SARS‐CoV‐2)	*In utero* exposure to maternal SARS‐CoV‐2 infection, neonatal SARS‐CoV‐2 infection, psychological stress	Duration of gestation, Cognitive and brain development; medical outcomes, Infant mortality	4 Studies,^b^ 2 Studies,^b^ 7 Studies,^a^ 6 Studies,^a^
Basilio et al. (2022)	10 Studies (*n* = 4,724,862)	Mainly retrospective cohort	Wildfire	Objective stress	Duration of gestation, Size and weight outcomes, Medical outcomes, Cognitive and brain development; sex‐ratio at birth	5 Studies,^a^ 7 Studies,^b^ 1 Study,^b^ *n* is not reported,^c^ 1 Study, fewer males
Navara (2010)	16 Studies, (*n* = not reported) only 3 on natural disasters	Retrospective and prospective studies are included. Comparative approach	Earthquake, flood, smog	Physiological measures, subjective stress, objective stress	Sex ratio at birth	3 Studies, fewer males
Fontanesi et al. (2024)	25 Studies (*n* = 3,115,563)	Retrospective and prospective studies are included. The majority are retrospective.	Earthquake, volcanic eruption, hurricanes, floods, epidemics and pandemics	Subjective stress, objective stress	Sex ratio at birth	25 Studies, fewer males
Matvienko‐Sikar et al. (2021)	16 Studies (*n* = 140,651), only 4 on natural disasters	8 Prospective cohort studies, 7 retrospective cohort studies, 1 cross‐sectional study	Flood, ice Storm,	Psychological stress, physiological stress, objective stress, timing of stress exposure	Size and weight outcomes,	4 Studies,^b^
Mallett and Etzel (2018)	141 (*n* = not reported)	Mainly retrospective	Floods	Reproductive health, exposure to moulds and reproduction, leptospirosis	Size and weight outcomes, Medical outcomes, Infant mortality,	5 Studies,^b^ *n* is not reported,^b^ 4 Studies,^a^
King et al. (2012)	Not reported	Mainly prospective	Ice storms, floods	Objective stress, physiological stress, subjective stress	Size and weight outcomes, Duration of gestation, Cognitive and brain development, behavioural outcomes, Medical outcomes	*N* is not reported,^b^ *N* is not reported,^b^ *N* is not reported,^b^ *N* is not reported,^a^ *N* is not reported,^b^

^a^ Mainly positive associations with prenatal maternal stress.

^b^ Mainly negative association with prenatal maternal stress.

^c^ Inconsistant results.

After the selection of the reviews and meta‐analysis, the quality of the selected studies was assessed by two of the authors independently. To assess meta‐analysis and systematic reviews, we used the Joanna Briggs Institute (JBI) Critical Appraisal tool (Whiting et al. 2003). For the assessment of the included narrative reviews, we used a scale for narrative review articles (SANRA) as most criteria of the JBI are not applicable for narrative reviews (Baethge et al. 2019). After the assessment, we compared the results of the scoring tools and discussed until consent for the scoring of each article was reached (the final scoring for each article is displayed in Tables 3 and 4).

**TABLE 3:** JBI critical appraisal for each selected systematic review and meta‐analysis.

Study	1. Review question	2. Inclusion criteria	3. Search strategy	4. Sources and resources	5. Appraising studies	6. Two researchers	7. Data extraction	8. Study combination	9. Publication bias	10. Policy/practice recommendations	11. Specific directives
Amjad et al. (2021)	Yes	Yes	Yes	Yes	Yes	Yes	Yes	Yes	No	Yes	Yes
Fontanesi et al. (2024)	Yes	Yes	Yes	Yes	Yes	Yes	Yes	Unclear	Yes	Unclear	Yes
Lamichhane et al. (2019)	Yes	Yes	Yes	Yes	Yes	No	Yes	Unclear	Yes	No	Yes
Lafortune et al. (2021)	Yes	Yes	Yes	Yes	Yes	Unclear	Yes	Yes	Yes	Yes	Yes
Matvienko‐Sikar et al. (2020)	Yes	Yes	Yes	Yes	Yes	Yes	Yes	Unclear	Yes	No	Yes

**TABLE 4:** SANRA score for each selected narrative review.

Study	1. Importance	2. Aims	3. Literature search	4. Referencing	5. Reasoning	6. Data presentation	Total
Basilio et al. (2022)	2	2	1	2	2	2	11/12
King et al. (2012)	2	2	1	2	2	2	11/12
Kyle and Dumitriu (2021)	2	1	0	2	1	1	7/12
Navara (2010)	2	2	0	2	2	2	10/12
Mallett and Etzel (2018)	2	2	1	2	2	1	10/12

### 2.1 Summary of the Results

The results of the selected meta‐analysis and reviews highlight consistent effects of PNMS on various areas of development. Thus, an open‐ended summary of all effects, which were reported in the selected studies, indicate effects of PNMS on six developmental areas. The reported effects in the six developmental areas can be summarized as follows (for a synthesis of the results, see below).

#### 2.1.1 Reduced Body Size and Weight

Seven of the selected reviews investigated the effects of PNMS due to natural disasters on weight outcomes (Amjad et al. 2021; Basilio et al. 2022; King et al. 2012; Lafortune et al. 2021; Lamichhane et al. 2020; Mallett and Etzel 2018; Matvienko‐Sikar et al. 2021). Amjad et al. (2021), Basilio et al. (2022), Mallett and Etzel (2018) and Matvienko‐Sikar et al. (2021) report that PNMS due to natural disasters is generally associated with lower birth weight in children. Moreover, King et al. (2012), Lafortune et al. (2021), Lamichhane et al. (2020), and Mallett and Etzel (2018) outline that exposure to a natural disaster during pregnancy is associated with a higher risk of child overweight and obesity later in life. Additionally, five of the articles point to effects of PNMS on head circumference and/or waist‐to‐height ratio. The reported effects are of different types but they all indicate an influence of PNMS on head circumference and/or waist‐to‐height ratio which both are relevant predictors for further child development. In detail, Lafortune et al. (2021) report that PNMS is positively correlated with head circumference and Lamichhane et al. (2020) report that PNMS is positively associated with subscalpular thickness, triceps thickness, waist‐to‐height ratio and fat‐mass index, which are indicators for overweight and obesity later in life. According to their review, Basilio et al. (2022) found that exposure to wildfire smoke leads to restricted foetal growth because of DNA methylation due to toxins in the air, which pass the placental barrier during pregnancy. Similarly, King et al. (2012) found that PNMS because of ice storms was related to smaller infant size, head circumference, and foetal growth (see also Laplante et al. 2008 for a study on cognitive and emotional effects). As reported in the review, Mallett and Etzel (2018) found that PNMS is associated with smaller head circumference at birth. Hence, PNMS can be seen to influence body growth and development in the long term by growth‐impeding effects at the beginning, which lead to unhealthy weight development later in life.

#### 2.1.2 Gestational Duration and Preterm Birth

Five reviews investigate gestational duration as an outcome variable affected by PNMS (Amjad et al. 2021; Basilio et al. 2022; King et al. 2012; Kyle and Dumitriu 2021; Lafortune et al. 2021). However, effects vary. Specifically, Basilio et al. (2022) concluded in their review that a majority of studies found a significant positive correlation between preterm birth and exposure to wildfire smoke. And King et al. (2012) as well as Kyle and Dumitriu (2021) report a positive correlation between prenatal exposure to a natural disaster and shorter gestation. Contrary to that, Lafortune et al. (2021) report that disaster related PNMS led to greater gestational length and Amjad et al. (2021) report inconsistent results. Therefore, various influences of PNMS on gestational length seem to work, resulting in various effects depending on other factors.

#### 2.1.3 Neural and Cognitive Development

Four of the reviews and meta‐analysis report an association between PNMS and cognitive and brain developmental outcomes of the child (Basilio et al. 2022; King et al. 2012; Kyle and Dumitriu 2021; Lafortune et al. 2021). Evidence synthesized by Lafortune et al. (2021) suggests a significant positive correlation between disaster related PNMS and the child's cognitive development as measured by their IQ, language abilities, developmental quotient, and playstyle. Similarly, King et al. (2012) report that while low levels of general PNMS can have positive effects on cognitive development, high levels of disaster related PNMS are correlated with poor language ability and lower IQ as well as a less mature playstyle compared to non‐exposed children at the age of 2. Additionally, Kyle and Dumitriu (2021) report a negative association between COVID‐19 related PNMS and the child's early language abilities and child‐mother bonding, arguably due to infant serotonin transporter gene methylation during pregnancy. Similarly, Basilio et al. (2022) propose that prenatal exposure to wildfire smoke can lead to negative neurodevelopmental outcomes. Specifically, they report that exposure to wildfire smoke leads to an increase in biomarkers typical for DNA damages and neurodevelopmental disorders later in life. Hence, due to various negative influences on the prenatal cognitive development, long‐term cognitive difficulties arise, which in turn complicate all subsequent developments—such as language development but also development of eloquent social as well as emotional capabilities—and can lead to serious illnesses.

#### 2.1.4 Emotional and Behavioural Development

Two of the selected studies investigated behavioural and emotional outcomes of children of mothers who were exposed to natural disasters (King et al. 2012; Lafortune et al. 2021). In both studies, internalizing (i.e., anxiety and depression) and externalizing (i.e., aggression) behaviours of children were assessed. According to these studies, PNMS was associated with higher levels of critical internalizing and externalizing behaviour problems of the children even later in life. Furthermore, they report that exposure to a natural disaster and the associated objective PNMS was significantly associated with subclinical and clinical symptoms of autistic behaviour of the child later in life.

#### 2.1.5 Medical Diseases and Infant Mortality

Four of the reviews and meta‐analysis investigate effects of PNMS on medical outcomes of children (Basilio et al. 2022; King et al. 2012; Kyle and Dumitriu 2021; Mallett and Etzel 2018). Basilio et al. (2022) report in their review that toxins of wildfire smoke are associated with inflammation, oxidative stress, endocrine disruption, and cellular dysfunction which are all contributors for poor birth outcomes. In detail, they conclude that children which have been prenatally exposed to wildfire smoke have a higher likelihood to suffer from epigenetic changes which decrease the number of proinflammatory T cells, exacerbate asthma symptoms and negatively affect various immune processes. Based on the data from their meta‐analysis, King et al. (2012) outlined that exposure to a natural disaster can lead to higher risk of metabolic diseases such as diabetes as well as asthma. Kyle and Dumitriu (2021) suggest that prenatal exposure to COVID‐19 was associated with fewer white blood cells, neutrophil and lymphocyte counts in newborns. However, they report that children exposed to COVID‐19 generally had only mild symptoms and a good immune response to the virus.

Additionally, two of the articles reviewed effects of exposure to a natural disaster and infant mortality (Amjad et al. 2021; Mallett and Etzel 2018). Amjad et al. (2021) found that exposure of pregnant women to wildfire smoke led to an overall higher child mortality, especially if the exposure took place in the third trimester of pregnancy. Similarly, Mallett and Etzel (2018) report that floods cause a higher pre and postnatal child mortality through various mechanisms, such as infectious diseases, maternal malnutrition during pregnancy, contaminated water and worse medical care.

#### 2.1.6 Sex Ratio at Birth

Besides infant mortality, three reviews mention the skewing of the sex ratio at birth after natural disasters (Basilio et al. 2022; Fontanesi et al. 2024; Navara 2010).

All three reviews report a significant correlation of exposure to natural disasters during pregnancy and the number of male offspring, indicating that after a natural disaster there are fewer male babies born.

### 2.2 Overlap in Results

In the published studies, there are overlaps which leads to an overrepresentation of certain natural disasters. In detail, four studies (King et al. 2012; Lafortune et al. 2021; Lamichhane et al. 2019; Matvienko‐Sikar et al. 2020) describe the effects of the snowstorm in Canada in 1998 and three studies describe the effects of the flood in Iowa in 2008 (Lafortune et al. 2021; Lamichhane et al. 2019; Matvienko‐Sikar et al. 2020). Thus the results may be seen as slightly biased towards the consequences of these disasters. However with that being said, in general, the degree of overlap between primary studies (and individuals in those studies) is minimal. This is also indicated by the Corrected Covered Area (CCA), a standard metric for quantifying overlap (using a citation matrix with rows representing unique studies and columns representing reviews). The resulting CCA was 1.9%, indicating only minimal overlap in primary studies across the included reviews and meta‐analyses.

### 2.3 Synthesis of the Results

**FIGURE 2:**
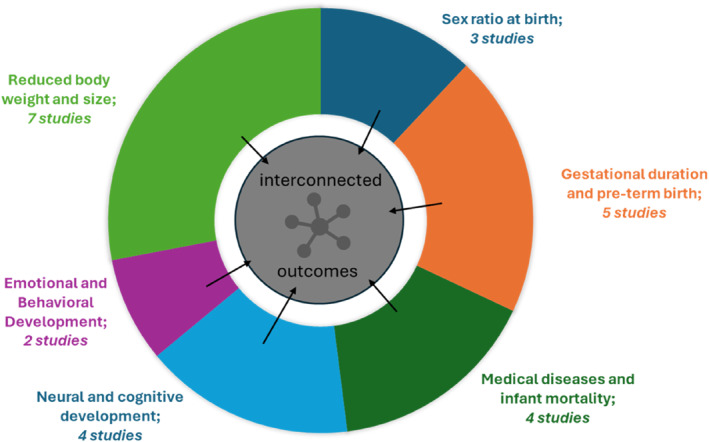
Frequency of outcome variables in the selected reviews and meta‐analysis. The number indicates the frequency for the inclusion of the variable as an outcome.

A synthesis of the reported results indicates, on the one hand, that PNMS causes negative effects on all relevant developmental areas. This is illustrated by the differently coloured areas in a pie chart in Figure 2. Strength‐of‐evidence varies for the negative effects in different developmental areas as indicated by the different numbers of studies reporting negative effects in the particular developmental area (see Figure 2, indicated by the number of studies and different sizes of the coloured areas). On the other hand, a synthesis of the reported results indicates the interdependence of the negative effects. Specifically, against the background of developmental steps that build on one another, far‐reaching effects of early developmental difficulties on later developmental processes are highlighted (as indicated by the arrows in Figure 2). In detail, the identified effects on gestational duration which mostly take the form of preterm birth indicate overall limited development conditions due to the shortened time. Overall limited development conditions mean impairments in physical development as well as impairments in neurological development. Further, the identified impairments in physical development as indicated by reduced body size and weight, favour the development of physical illnesses and the identified impairments in neurological development favour restricted cognitive development. Restricted cognitive development then has far‐reaching consequences, including significantly impaired language acquisition. Limited language skills then mean significantly limited opportunities to acquire important social skills and emotional abilities. This again favours the emergence of critical internalizing and externalizing behaviour, that is, reduced expression of needs, that is being highly introverted or acting out aggressively and loudly. Behaviour like this per se indicates a child's emotional stress. Moreover, it favours the development of mental illness later in life.

Notably, one study investigated the effects of COVID‐19. While the pattern of results is comparable to other studies and disasters, COVID‐19 as a disaster sticks out because it is not a nature‐based and physical disaster like a blizzard or an earthquake. At the time of writing, COVID‐19 is a fairly recent disaster and the full effects of this prenatal stressor on birth outcomes and developmental trajectories cannot yet be fully understood.

## 3 Discussion

The here reported systematic review of research findings about the connection of PNMS and a child's development and psychological health confirms again that maternal stress during pregnancy has far‐reaching consequences for the child. The summary of published empirical findings in this field indicates that PNMS plays an important role in the child's development by significantly influencing the size and weight of the child, the duration of pregnancy, the neural and cognitive development of the child, the emotional and behavioural development of the child as well as whether any diseases were present at birth or even child mortality. Moreover, the synthesis of the empirical findings highlights that PNMS will probably cause long‐lasting negative effects for the unborn child because early damage to even single developmental stages also impedes subsequent developmental stages.

Importantly, this umbrella review might have several limitations worth considering. Firstly, no study protocol was registered a priori for this review. Due to the search strategy used by the authors, a potential language and publication bias should be considered at this point, as non‐English language studies and grey literature were excluded. Additionally, only the two most relevant databases were considered for analysis, which may have led to the omission of other reviews that are not listed in those databases. However, given the large overlap between the two databases and the additional forward‐backward search of the articles included this risk is limited. Second, this review might inherit methodological flaws from the included meta‐analyses and systematic reviews, potentially compromising the overall reliability of findings. Third, the inclusion of studies with diverse designs, populations, exposure definitions, types of disasters, and outcome measures can reduce the comparability of results. The generalizability of the results is limited due to the predominance of studies conducted in high‐income countries. Fourth, umbrella reviews are susceptible to publication bias, as studies reporting significant effects are more likely to be published, which may skew the findings in a direction that may exaggerate the effects of natural disasters on PNMS and their consequent effects on child development. Additionally, when investigating the stress experienced by mothers after natural disasters, it must be taken into account that although natural disasters have become a more frequent subject of study due to climate change, they occur so rarely that there were not enough different incidents for the authors to consider in their research. Some meta‐analyses and reviews included in this umbrella review may incorporate overlapping primary studies, leading to potential double‐counting and an overestimation of effects. However, the CCA analysis showed that overall there was only a 1.9% overlap between all studies. Lastly, because this review synthesizes observational data from multiple sources, establishing causality remains a challenge. Reported associations may be influenced by confounding factors, residual bias, or variations in analytical approaches across studies. However, the reported studies here have been chosen without any personal bias, because the authors of the current review are not authors of empirical studies in this field. Additionally, investigating the effects of PNMS due to exposure to natural disasters helps in isolating the effects of stress from person‐specific parameters as best as possible since natural disasters affect broad populations simultaneously and the exposure to this particular stressor is less confounded with person‐specific factors than the exposure to other stressors.

This being said and kept in mind, the here reported findings provide a basis for important discussion points. First, as already revealed by previous studies, the synthesis of the included results indicates that the way maternal stress influences the unborn child varies. This is probably linked ‐ on the one hand ‐ to varying contextual factors which cause a variety in the way a natural disaster influences people. Thus, the negative effects of stress due to natural disasters most probably vary between different countries with for example different health care systems. Moreover, as already mentioned in the introduction, exposure to natural disasters depends on person‐specific factors meaning that stress is perceived and coped differently by different people. Consequently, stress is perceived and coped differently by different mothers and also by different children ‐ based on their individual developmental processes. Specifically, there are various stress phenotypes meaning that people differ in the way they experience stress and resilience factors in pregnancy such as social support and coping mechanisms seem to play a major role in experiencing stress (Walsh et al. 2019). It is also important to consider social determinants of health (SDoH; Solar and Irwin 2010). These can influence behaviour directly or indirectly, but in any case moderate the consequences caused by natural disasters in the mother's stress experience. Economic resources, education, discrimination, and social support can increase or mitigate the emotional consequences and must be considered as individual and structural components (Braveman et al. 2011). In this regard, the finding of an overlap of the negative effects of PNMS on the foetal development as indicated by the here reported results highlights a rather basic effect of prenatal stress. Specifically, besides various contextual and person‐specific factors, effects of PNMS on the same developmental areas were found in various studies (Figure 2).

Further, the extent to which and the way in which PNMS impairs foetal development seems to depend on the timing of stressor exposure during pregnancy. On the one hand, the sensitivity to psychobiological stress seems to decrease as pregnancy progresses (Entringer et al. 2010), suggesting that stress in early pregnancy has greater impact (Glynn et al. 2001). In line with that, empirical findings indicate that early pregnancy elevated cortisol levels were related to altered amygdala volumes in female children at the age of seven, but elevated cortisol levels in mid or late pregnancy were not (Buss et al. 2012). On the other hand, the saturation of the enzyme 11ßHSD‐2 in the placenta is decreased in late pregnancy compared to the earlier pregnancy stages and the resulting physiological increase in the endogenous glucocorticoid cortisol stimulates foetal organ maturation processes (Braun 2020; Murphy and Clifton 2003; Mohammed 2012). And at a decreased 11ß‐HSD‐2 level, heightened foetal vulnerability to the effects of cortisol is suggested (Rakers et al. 2020). In line with that, negative effects of PNMS on children's motor development were found if the stressor occurred in mid (Buss et al. 2010; Diego et al. 2005; Field et al. 2006) and late pregnancy (Moss et al. 2017; Simcock et al. 2016). Thus, it seems that stress, regardless of the timing, is harmful for the mother as well as for the child. However, the effects of PNMS vary systematically. In detail, PNMS harms the development taking place. In terms of a neural basis which has to be formed first, neural development happens earlier than for example motor development. Hence, PNMS during early pregnancy has greater impact on neural development such as amygdala growth, whereas PNMS during later pregnancy has greater impact on motor skills—again highlighting the interdependence of developmental stages also in the womb.

### 3.1 Resulting Recommendations

By highlighting the potential negative consequences of PNMS for all developmental areas and the impairment of subsequent developmental steps conclusions can be drawn about specific recommendations for protective interventions. Importantly, to the best of our knowledge, those recommendations do not have to be limited or focussed on interventions for the case of a natural disaster. Rather, the here reported results depict effects of PNMS as best as possible independent of person‐specific factors in order to achieve a potentially high external validity. Nevertheless, authors have made recommendations on how to improve support for pregnant women in crisis situations. In particular, medical professionals working in disaster relief should be trained in the areas of maternal mental health and PNMS. Support programmes should include access to psychological support for women in the perinatal period in order to mitigate long‐term negative effects on their children (Sato et al. 2016). Psychological first aid according to WHO guidelines (World Health Organization and War Trauma Foundation and World Vision and International 2011) and cognitive behavioural therapy (CBT) are possible interventions at the population and individual level to reduce PNMS due to natural disasters (see King et al. 2021).

In line with etiological models, predictive adaptive response, and the foetal programming hypothesis, the here depicted results highlight aspects of pathogenesis but also of salutogenesis. First, the reported results highlight various negative impacts of PNMS for the child's health, which goes in line with theories like the ‘developmental origins of health and disease’ theory (DOHaD; Gillman 2005). According to the DOHaD hypothesis, both a child's development and susceptibility to disease later in life can be determined by environmental influences even before birth. PNMS, caused by a natural disaster, is an environmental factor that can affect foetal development during a sensitive phase. However, DOHaD traditionally focuses on the impacts of chemical exposures (e.g., air pollutants) or (mal)nutrition on health outcomes and the here reported results indicate that interactions of various factors should be taken into account. Thus, research should reflect real‐world scenarios by examining the interactions of multiple exposures including chemicals, nutrition, stress, infections, and drugs (Haugen et al. 2015) and should explore the broader impact of environmental chemicals on various organs and disease syndromes rather than focusing on single chemical‐disease relationships. As such, the assessment of natural disasters provides a possibility to assess a multi‐factorial stressor and thereby to fully grasp the integrated toxicity of exposures.

There also lies an opportunity in considering that a child's development can already be severely impaired during pregnancy. Considering the previously reported results, necessary assistance can be two‐folded. First, maternal stress should be addressed during prenatal checkups at hospitals or by gynaecologists as well as in midwife consultations or childbirth preparation courses. Additionally, Hentges et al. (2019) suggest a preventive program that can lower prenatal stress and lasts into the postnatal period so that negative effects are counteracted in the best possible way both prenatally and postnatally.

A second important chance in knowing about highly possible negative effects of PNMS lies in the possibility to address potential developmental difficulties after birth. Specifically, by knowing about maternal stress as a risk factor for foetal development and especially by knowing about the interdependence of the negative effects, a significant chance lies in targeted development support for the child after birth. Hence, the knowledge that a child's development may already be in danger during pregnancy is necessary to ensure that the resulting needs of the child can be taken into account after birth. Moreover, by focussing on changes in early development targeted early intervention is able to prevent later physical and mental diseases. In concrete terms, specific developmental support can be provided to the child if the extent to which its development was already restricted before birth is taken into account specifically. In this respect, knowledge of possible negative influences even before birth is essential to give the child the developmental support it needs so that developmental impairments can be counteracted in the best possible way. In this regard, one possibility also lies in sensitive and responsive parental care. Thus, sensitive and responsive parental care ‐ also by a non‐related but reliable caregiver ‐ is a powerful resilience factor. It helps the child to cope with upcoming developmental tasks (Hohm et al. 2017) and current reviews also indicate epigenetic, neurobiological, and behavioural consequences of sensitive and responsive maternal care claiming a prenatal‐postnatal interplay in shaping the child's development (Monk et al. 2012). Taken together, by addressing potential congenital developmental difficulties, the vicious circle of negative effects as depicted in Figure 2 can be interrupted and thereby otherwise occurring long‐lasting effects can be prevented.

## Author Contributions

All authors were involved in the conceptualization, with KB and SS providing the initial ideas. KB, LH, HW conducted the literature search. All authors were involved in analysis. All authors contributed to the first draft of the manuscript and were involved in copy‐editing. SS and MF had supervisory roles.

## Conflicts of Interest

The authors declare no conflicts of interest.

## AI Statement

Generative AI was not used to generate parts of this manuscript. Software was used to improve the readability and language of the manuscript (e.g., checking for grammar).

## Supplemental Material

Please refer to https://osf.io/rd2xm/?view_only=5f7ab79ca1bf439ba5c878127c1aaa76.

## Data Availability

Data sharing not applicable to this article as no datasets were generated or analysed during the current study.
